# Pigmentierter Lidtumor unklarer Dignität

**DOI:** 10.1007/s00347-020-01132-3

**Published:** 2020-07-03

**Authors:** Friederike Dorothee Bosche, Ralph-Laurent Merté, Dieter Metze, Nicole Eter, Natasa Mihailovic

**Affiliations:** 1 Klinik für Augenheilkunde, Universitätsklinikum Münster, Domagkstr. 15, 48149 Münster, Deutschland; 2 Klinik für Hautkrankheiten, Universitätsklinikum Münster, Münster, Deutschland

## Anamnese

Eine 75-jährige indonesische Patientin stellte sich mit der Bitte um Mitbeurteilung bei unklarem pigmentiertem Tumor des rechten lateralen Lidwinkels in unserer Klinik vor. Die Läsion sei vor einigen Jahren erstmals auffällig geworden und sei langsam größenprogredient. Anamnestisch seien keine ophthalmologischen und systemischen Vorerkrankungen bekannt. Die Patientin würde lediglich regelmäßig Tränenersatzmittel nutzen.

## Klinischer Befund

**Figure 1:**
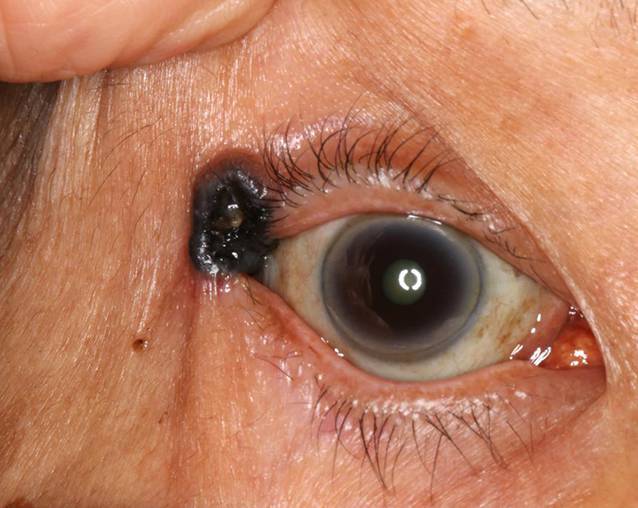

**Figure 2:**
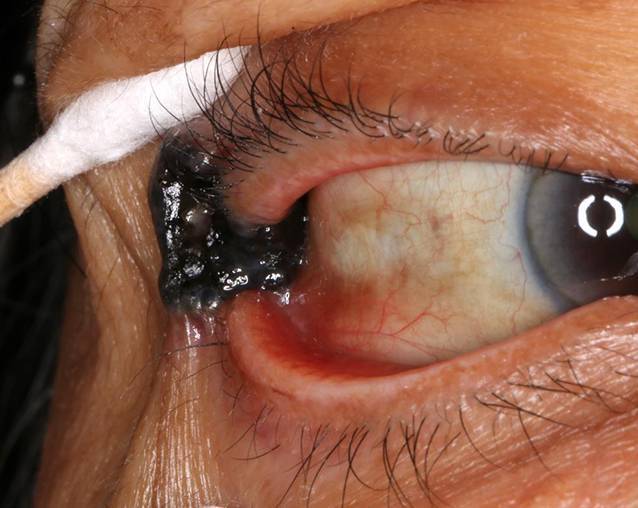

Am lateralen Lidwinkel des rechten Auges zeigte sich ein intensiv pigmentierter, zentral ulzerierender, teilvaskularisierter Tumor mit verwaschenen Rändern und erhabenem Randwall (Abb. 1). Die Ausbreitung erstreckte sich über den lateralen Kanthus bis auf die tarsale Konjunktiva (Abb. 2). Eine Beteiligung der bulbären Konjunktiva konnte initial nicht sicher ausgeschlossen werden. Zudem zeigte sich spaltlampenbiomikroskopisch beidseitig eine Melanosis conjunctivae, ein Arcus lipoides und eine beginnende Katarakt bei intraokular ansonsten reizfreiem Befund. Fundoskopisch zeigte sich ein unauffälliger Befund der Netzhaut inklusive der Peripherie. Die Augenmotilität war beidseits frei.

## Weiteres Procedere

Bei differenzialdiagnostischem Verdacht auf ein malignes Melanom erfolgte parallel zur Planung einer chirurgischen Tumorexzision die Initiierung eines Stagings inklusive kontrastmittelgestützter Magnetresonanztomographie des Schädels (cMRT). Hierbei zeigte sich eine ausgedehnte periorbitale Weichteilschwellung rechts mit begleitender Sinusitis maxillaris. Morphologisch war der cMRT-Befund primär mit einer entzündlichen Genese vereinbar, die Maskierung eines malignen Melanoms konnte jedoch nicht ausgeschlossen werden. Eine klinische dermatologische Untersuchung ergab keinen Anhalt für eine weitere kutane maligne Neoplasie.

## Wie lautet Ihre Diagnose?

## Histologie

Mikroskopisch zeigte sich in dem 1,2 ×1,0 cm großen Exzidat eine von der Epidermis ausgehende knotige, ulzerierte Proliferation basaloider, zum Teil pigmentierter Zellen mit deutlicher Spaltbildung zum umliegenden muzinreichen Bindegewebe. Die Tumortiefe lag bei 3 mm, der Tumor erreichte die quergestreifte Muskulatur des M. orbicularis und grenzte zudem an einem Rand an die konjunktivale Schleimhaut (Abb. 4). Insgesamt entsprach der Befund damit einem nodulären Basalzellkarzinom (BCC).

## Definition

Das BCC ist mit einer Inzidenz von 200 pro 100.000 Einwohner jährlich der häufigste maligne Tumor des Menschen in Mitteleuropa und tritt meist im Kopf- und Halsbereich auf. Von den malignen Augenlidtumoren macht das BCC 86 % der Fälle aus. Metastasen sind nur in bis zu 0,55 % der Fälle beschrieben [4].

Etwa 2,3–8,5 % der BCC sind klinisch sichtbar pigmentiert, und sie treten, anders als nicht pigmentierte BCC, vermehrt bei dunkleren Hauttypen auf [3, 7]. Primäre BCC der Karunkel sind sogar in bis zu 45 % der Fälle pigmentiert [4, 8]. Aufgrund der irregulären Form und des dunklen Pigments können pigmentierte BCC leicht mit malignen Melanomen verwechselt werden. Insgesamt haben pigmentierte BCC ein eher fleckiges Erscheinungsbild mit oberflächlicher lokalisiertem Pigment, wohingegen Melanome intensiveres und maskierendes Pigment aufweisen [6]. In unserem Fall war das BCC allerdings auch sehr intensiv pigmentiert. Weitere dermatoskopische Aspekte eines pigmentierten BCC sind baumartige Teleangiektasien, Perlmuttglanz und große blaugraue eiförmige Areale. Beim nodulären BCC treten Ulzerationen gehäuft auf. Es wurden bisher keine biologischen oder Verhaltensunterschiede zwischen pigmentierten und nicht pigmentierten Basalzellkarzinomen dokumentiert [3]. Neben dem malignen Melanom sind weitere Differenzialdiagnosen des pigmentierten BCC pigmentierte, blaue Nävi, pigmentierte seborrhoische Keratosen sowie pigmentierte Plattenepithelkarzinome und Plattenepithelpapillome [3].

## Therapie und Verlauf

**Figure 3:**
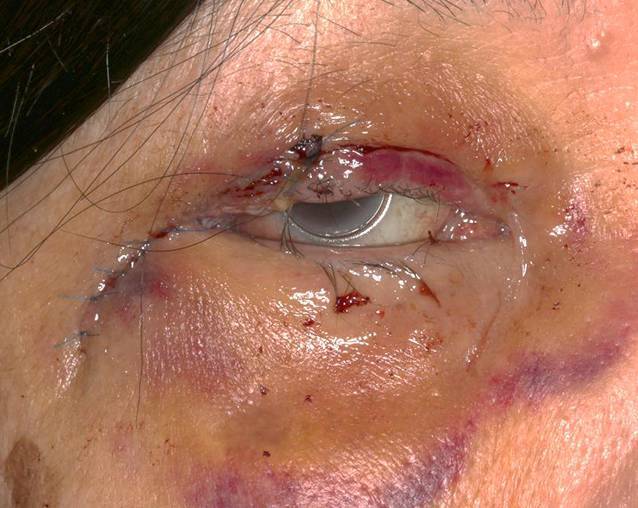

Nach der histologischen Sicherung einer Exzision im Gesunden (R0) erfolgte wenige Tage im Anschluss die plastische Deckung. Die Rekonstruktion des lateralen Kanthus erfolgte mittels eines zweigeteilten, gestielten Periostlappens, wobei der obere Stiel zur Rekonstruktion des lateralen Oberlids, der untere Stiel zur Rekonstruktion des lateralen Unterlids genutzt wurde. Der Hautdefekt konnte mit einem gestielten Hauttransplantat aus dem ipsilateralen Oberlid gedeckt werden. Die Abb. 3 zeigt den postoperativen Befund 9 Tage nach der plastischen Deckung.

**Figure 4:**
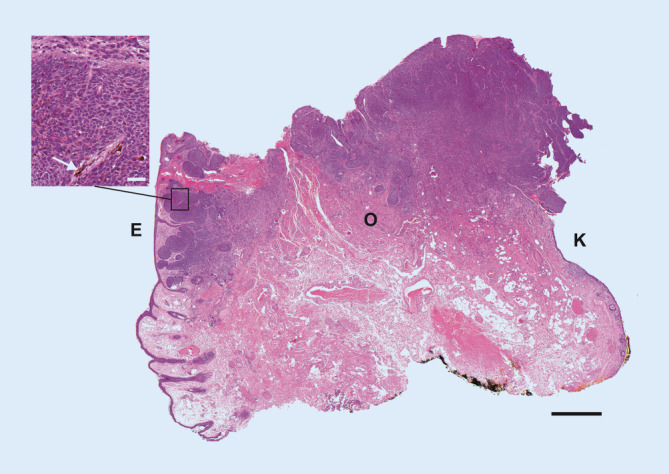

Die histologisch kontrollierte, vollständige Resektion im Gesunden ist beim BCC, sofern möglich, die Therapie der Wahl [2]. Bei niedrigem Rezidivrisiko (nodulärer Subtyp, gut definierte Begrenzung, maximaler Tumordurchmesser am Augenlid <6 mm) sollte ein Sicherheitsabstand von 3–5 mm eingehalten werden. Bei hohem Rezidivrisiko (infiltrativ wachsend, maximaler Tumordurchmesser am Augenlid >6 mm) sollte der Resektionsabstand mindestens 5 mm betragen, wenn keine mikroskopische Kontrolle möglich ist [5]. In unserem Fall betrug die Ausdehnung des BCC im zentralen Gesichtsbereich zwar >6 mm, gleichzeitig ist das Rezidivrisiko bei nodulären und pigmentierten BCC aber niedrig [5]. Laut der S2k-Leitlinie „Basalzellkarzinom“ kann gerade bei pigmentierten BCC aufgrund ihrer guten Abgrenzbarkeit ein geringerer Resektionsabstand gewählt werden [5]. Generell ist am Lid jeder Millimeter erhaltenes gesundes Restgewebe für die spätere Rekonstruktion entscheidend [9]. In unserem Fall wurde daher insbesondere unter Berücksichtigung einer Beteiligung des Kanthus und der tarsalen Konjunktiva ein Sicherheitsabstand von 3 mm gewählt.

**Diagnose:** Pigmentiertes noduläres Basalzellkarzinom (BCC)

Bei Kontraindikationen für eine Operation oder Inoperabilität kann eine Strahlentherapie, eine Therapie mit Vismodegib [1], eine photodynamische Therapie oder bei superfiziellen Basalzellkarzinomen eine topische Therapie mit 5‑Fluorouracil 5 % oder Imiquimod 5 % erwogen werden. Diese Alternativen sind der operativen Resektion jedoch hinsichtlich der lokalen Befundkontrolle unterlegen [5].

Eine Nachsorge zur Früherkennung von Lokalrezidiven wird bei niedrigem Rezidivrisiko nach 6 Monaten und anschließend jährlich empfohlen. Bei hohem Rezidivrisiko ist eine Nachsorge initial alle 3 Monate sinnvoll. Unsere Patientin wollte die Nachsorge heimatnah durchführen lassen.

## Fazit für die Praxis

- Ein pigmentiertes Basalzellkarzinom stellt eine Differenzialdiagnose zum malignen Melanom dar.
- Goldstandard ist die histologisch schnittrandkontrollierte Resektion im Gesunden. Hierbei ist der empfohlene Mindestabstand unter Berücksichtigung der lokalen anatomischen Verhältnisse (Liddefekte) zu bedenken.
- Pigmentierte Lidtumoren können neben Basalzellkarzinomen und malignen Melanomen auch pigmentierte, blaue Nävi, pigmentierte seborrhoische Keratosen, sowie pigmentierte Plattenepithelkarzinome und Plattenepithelpapillome darstellen.
